# Zika virus disease, microcephaly and Guillain-Barré syndrome in Colombia: epidemiological situation during 21 months of the Zika virus outbreak, 2015–2017

**DOI:** 10.1186/s13690-017-0233-5

**Published:** 2017-11-02

**Authors:** Nelson Méndez, Misael Oviedo-Pastrana, Salim Mattar, Isaac Caicedo-Castro, German Arrieta

**Affiliations:** 1 Instituto de Investigaciones Biológicas del Trópico, Universidad de Córdoba, Carrera 6 #, 76-103 Montería, Córdoba Colombia; 2 Facultad de Ingeniería, Departamento de Ingeniería de Sistemas y Telecomunicaciones, Universidad de Córdoba, Montería, Colombia; 3 Corporación Universitaria del Caribe (CECAR), Grupo de Salud Pública, Km 1, vía Corozal, Sincelejo, Colombia; 4 Clínica Salud Social, Carrera 16 # 27A, -74 Sincelejo, Colombia

**Keywords:** Infectious diseases vectors, Epidemiology, Culicidae, Morbidity, Nervous system congenital abnormalities, Education, Public health professional, Population

## Abstract

**Background:**

The Zika virus disease (ZVD) has had a huge impact on public health in Colombia for the numbers of people affected and the presentation of Guillain-Barre syndrome (GBS) and microcephaly cases associated to ZVD.

**Methods:**

A retrospective descriptive study was carried out, we analyze the epidemiological situation of ZVD and its association with microcephaly and GBS during a 21-month period, from October 2015 to June 2017. The variables studied were: (i) ZVD cases, (ii) ZVD cases in pregnant women, (iii) laboratory-confirmed ZVD in pregnant women, (iv) ZVD cases associated with microcephaly, (v) laboratory-confirmed ZVD associated with microcephaly, and (vi) ZVD associated to GBS cases. Average number of cases, attack rates (AR) and proportions were also calculated. The studied variables were plotted by epidemiological weeks and months. The distribution of ZVD cases in Colombia was mapped across the time using Kernel density estimator and QGIS software; we adopted Kernel Ridge Regression (KRR) and the Gaussian Kernel to estimate the number of Guillain Barre cases given the number of ZVD cases.

**Results:**

One hundred eight thousand eighty-seven ZVD cases had been reported in Colombia, including 19,963 (18.5%) in pregnant women, 710 (0.66%) associated with microcephaly (AR, 4.87 cases per 10,000 live births) and 453 (0.42%) ZVD associated to GBS cases (AR, 41.9 GBS cases per 10,000 ZVD cases). It appears the cases of GBS increased in parallel with the cases of ZVD, cases of microcephaly appeared 5 months after recognition of the outbreak. The kernel density map shows that throughout the study period, the states most affected by the Zika outbreak in Colombia were mainly San Andrés and Providencia islands, Casanare, Norte de Santander, Arauca and Huila. The KRR shows that there is no proportional relationship between the number of GBS and ZVD cases. During the cross validation, the RMSE achieved for the second order polynomial kernel, the linear kernel, the sigmoid kernel, and the Gaussian kernel are 9.15, 9.2, 10.7, and 7.2 respectively.

**Conclusions:**

This study updates the epidemiological analysis of the ZVD situation in Colombia describes the geographical distribution of ZVD and shows the functional relationship between ZVD cases and GBS.

## Background

In recent decades, Colombia has been an exceptional witness to an avalanche of emerging arboviruses. Outbreaks of Dengue, Venezuelan equine encephalitis, Chikungunya virus -associated arthralgia- and most recently Zika virus disease (ZVD) have caused significant health burden in Colombia. Other emerging viruses have been detected with unknown disease burden, such as West Nile virus, St. Louis encephalitis virus and hantavirus [1]. Guillain-Barre Syndrome (GBS), microcephaly and encephalitis due to arboviruses are novel and emerging public health issues in Colombia [2].

By March 10, 2017, autochthonous cases and vector-borne transmission of ZVD have been confirmed in 35 countries and 13 regions of the Americas [3]. In Latin America, the countries with higher ZVD cases rate (i.e., [autochthonous suspected + autochthonous confirmed]/100,000 pop) are Honduras (396), Belize (239.62), Colombia (221.29), Venezuela (197.72), El Salvador (187.44) and Brazil (168.09) [3, 4]. In Colombia and Latin American, during the last months, the infection with ZVD has had a huge impact on public health and its effects continue to be felt [2].

In October 2015, the first nine cases of ZVD in Colombia were reported in Turbaco (Bolivar State), in the Caribbean area [5]. So far, June 24th, 2017 and nearly 21 months later the disease has spread to the entire country where *Aedes aegypti* is established. Similar to other countries affected by Zika virus, the increases of congenital syndromes including microcephaly cases and GBS appeared abruptly coincident with the ZVD outbreak [6–12].

In Colombia, the National Health Institute (INS) conducts intensified surveillance for microcephaly and GBS cases associated with ZVD [13]. As a result, the INS has published preliminary reports about ZVD cases [8], GBS [8] and microcephaly cases associated with ZVD [8], other reports about transmission and epidemiology of Zika virus in Colombia have also been published [9–11]. In this study, we analyze the epidemiological situation of ZVD and its association with microcephaly and GBS in Colombia during a 21-month period, from October 2015 to June 2017.

## Methods

### Type of study and population

We conducted a retrospective descriptive epidemiological study of people with symptoms of ZVD reported by INS. The study included 21 months (from epiweek 40 beginning 10th October 2015, until epiweek 24, beginning 25th June 2017) of notifications of ZVD cases in 37 reporting administrative units of Colombia (32 states and 5 districts).

### Data collection and study variables

The public and freely available weekly epidemiological datasets from the National Health Institute of Colombia were used [13–15]. We imported these datasets into Microsoft Excel, where it was organized by notification areas (States and Districts). Then, from the accumulated cases of each week, we estimated the cases of the previous one. The variables studied were: (i) ZVD cases, (ii) ZVD cases in pregnant women, (iii) laboratory-confirmed ZVD in pregnant women, (iv) ZVD cases associated with microcephaly, (v) laboratory-confirmed ZVD associated with microcephaly, and (vi) ZVD associated to GBS cases.

For the present study, ZVD cases comprise the patients who were notified to the INS with symptoms of ZVD (fever and rash, and one or more of the following symptoms which are not explained by other medical conditions: non-purulent conjunctivitis, arthralgia’s, myalgia, headache or malaise), with and without laboratory confirmation. Laboratory-confirmed ZVD was defined as the presence of clinical symptoms of ZVD and a positive result for Zika virus RNA on RT-PCR assay [2, 9, 13]. A ZVD cases associated with microcephaly is any product of gestation (abortions, voluntary interruptions of pregnancy, stillbirths and live births) up to 12 months of age, which presents with a present a structural or functional alteration of the central nervous system, like microcephaly and others abnormalities of central nervous system, occurring during the gestation of a woman with symptoms of ZVD, with or without laboratory confirmation [2, 8]. A ZVD associated to GBS cases, is a person with laboratory-confirmed ZVD or with symptoms of ZVD, living in an endemic area of residence or travel within 15 days of symptom onset to an area with laboratory-confirmed Zika virus (ZIKV) circulation [7, 14, 15].

Population data and trends of Colombian population 2015–2017 were obtained by DANE (Statistics and data of Colombian government) [16]. The trends of live births ≤1 years was used as estimator of indirect of pregnant women and the data of live births was also taken from DANE [17, 18].

### Data analysis

#### Descriptive analysis of epidemiological situation of ZVD

ZVD transmission was analyzed by plotting the above mentioned variables against epidemiologic week/month in Microsoft Excel. The distribution of ZVD cases per gender/groups of age and the distribution per states were presented as percentage and attack rates (per each 100,000 population). Microcephaly cases were presented as a proportion over the total of live births during the study period, besides, GBS cases were presented as a proportion over the total ZVD cases.

### Geographic distribution of ZVD cases

A spatial analysis was conducted by importing the dataset and shapefiles of Colombian maps [19] into QGIS 2.14.3 (Development Team - Open Source Geospatial Foundation Project, 2016). With this analysis the goal was to describe the geographical distribution of ZVD cases (based on notification areas) and show areas with high concentration of cases or high intensity of cases per unit of surface. In order to assess the intensity of incidence per unit area (km^2^) we applied the density kernel technique, this technique produce a smoothed surface with the representation of disease distribution [19]. We used a bandwidth of 120 km. The maps were visualized for each trimester of each year and a summarized map for the entire period of study; moreover, to see the intensity of Zika incidences in each map, we established seven categories using as criteria the classification per quantile, the two highest categories show the clusters.

### Functional relationship between ZVD indicator and GBS cases

Kernel ridge regression (KRR) was used to estimate the number of GBS cases given the number of ZVD cases. We use this machine learning model with the kernel trick to disclose the functional dependence between the target variable, i.e., the number of GBS cases, and the independent observable variable, i.e., the number of ZVD cases.

In Fig. 3a and b, are depicted the number GBS cases (the target variable) vs ZVD cases (the independent variable), as we can see there is no linear relation among both variables, then a linear regression model underfits (Fig. B), It will fail in the prediction with high error. In such case, there are two options: firstly, we directly map the independent variables into a higher dimensional space, thereafter, the linear ridge regression is applied. The second option is adopting the kernel trick, which allows us to solve the regression problem by applying only inner product between independent variables without mapping them, thereby, the kernel trick is computationally less costly than the first option, because the original space of independent variables has less dimensions than the higher dimensional space of mapped variables. Therefore, we adopted KRR as a regression model. Saunders et al. [20], provide a broader explanation of this model.

Moreover, KRR solves the regression problem in an analytic fashion instead of an algorithmic one. The latter implies tuning more parameters than the analytic solution, e.g., the learning rate and the maximum number of iterations in the gradient descent algorithm. Furthermore, we used the R language and the CVST package to implement, tune, and test the KRR model.

On the other hand, in order to set up the parameters and choose the kernel function, we carried out the 5-fold cross validation technique, where the data set was randomly split in 5 folds, four of them for training and one fold for validation. This was done five times, changing the elements contained into each fold. The accuracy of the regression model was measured through the Root Mean Square Error and the coefficient of determination as well.

## Results

### Descriptive analysis of epidemiological situation of ZVD

From 10th October 2015 to 24th June 2017, 108,087 ZVD cases were notified by INS, Table 1 shows the distribution of gender and age groups. 66.2% of cases occurred in females, the attack rate was higher among women (286 per 100,000 population) than men (149 per 100,000 population). The highest AR was seen in the groups 25–29 years (375 per 100,000 population), 30–34 years (365 per 100,000 population), 20–24 years (326 per 100,000 population) and 35–39 years (305 per 100,000 population), representing 49.0% of the total cases (Table 1).

**Table 1:** Attack rates of Zika virus disease cases, by gender and age groups, Colombia, October 10th, 2015-June, 24th 2017

Variables	Categories	^a^Colombian Population	^b^ZVD cases	%	Attack rate per 100,000 population
Gender	Females	24,678,754	70,478	66.2	286
	Males	24,068,820	35,977	33.8	149
Age groups (years)	0–4	4,334,955	7705	7.2	178
	5–9	4,264,211	4465	4.2	105
	10–14	4,268,304	5146	4.8	121
	15–19	4,322,001	8495	8.0	197
	20–24	4,301,820	14,009	13.2	326
	25–29	4,021,285	15,072	14.2	375
	30–34	3,607,245	13,160	12.4	365
	35–39	3,263,097	9946	9.3	305
	40–44	2,916,896	7085	6.7	243
	45–49	2,871,857	6210	5.8	216
	50–54	2,728,948	5393	5.1	198
	55–59	2,302,860	3868	3.6	168
	60–64	1,801,943	2522	2.4	140
	≥65	3,742,153	3379	3.2	90

^a^Average population between 2015 and 2017

^b^ *ZVD* Zika virus disease

**Fig. 1:**
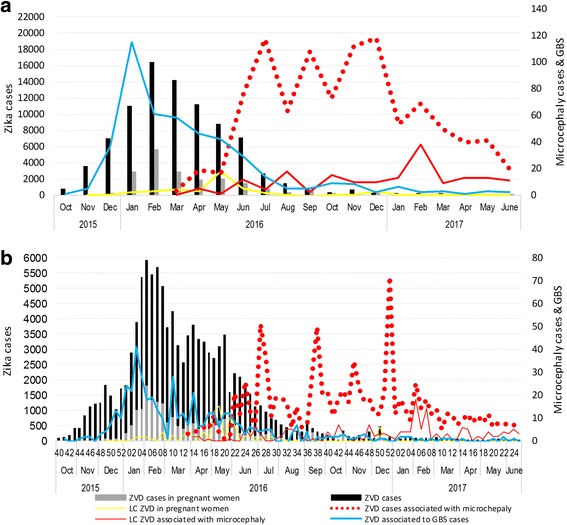
**a** Colombia, monthly distribution of Zika virus disease (ZVD) cases, microcephaly cases and Guillain-Barré Syndrome, 2015–2017. **b** Colombia, weekly distribution of ZVD cases, microcephaly cases and Guillain-Barré Syndrome, 2015–2017. In both figures, the left axis represents the ZVD cases, ZVD cases in pregnant women and laboratory-confirmed (LC) ZVD in pregnant women; the right axis represents the ZVD cases associated with microcephaly, laboratory-confirmed ZVD associated with microcephaly and ZVD associated to GBS cases

Monthly and weekly distribution of ZVD cases (*n* = 108,087), including 19,963 (18.5%) in pregnant women, 710 (0.66%) associated with microcephaly (4.87 cases per 10,000 live births), and 453 (0.42%) ZVD associated to GBS cases (41.9 GBS cases per 10,000 ZVD cases) are depicted in Fig. 1. During this period of time, 9802 (9.0%) ZVD cases was confirmed by ZIKV-specific RT-PCR, 6365 (5.91%) in pregnant women and 174 (0.16%) in cases of microcephaly (1.2 microcephaly cases per 10,000 live births).

Outbreak was first reported in October, 2015 (epiweek 40), in 2016 presented a largest number of ZVD cases (87.8%; 94,947 cases), and currently in 2017 (epiweek 1–25, January to June), have a median of 52 cases (range 15–123) (Fig. 1). The peak of the ZVD cases and ZVD cases in pregnant women was in February 2016 (epiweek 5–8), and was preceded by an increase of GBS cases during the period of November 2015 to January 2016 (peak of GBS in January 2016) (Fig. 1). Five months after the outbreak started, the microcephaly cases were increased in association with ZVD cases in the country (Fig. 1),

Table 2 shows population distribution of ZVD and ARs in Colombia, pregnant women diagnosed by laboratory are mainly concentrated in Norte de Santander, Huila, Meta, Casanare, Arauca, Caquetá, Tolima, Santander and Valle del Cauca.

**Table 2:** Attack rates per 100,000 population distribution of Zika virus disease in the total Colombian population and pregnant women, October 10th, 2015-June, 24th 2017

States	Total population					Pregnant women population				
	Population 2015–2017	ZVD cases	AR	^a^ LC ZVD cases	AR	Population 2015–2017	ZVD cases	AR	LC ZVD cases	AR
Amazonas	77,093	346	449	28	36	2157	42	1947	3	139
Antioquia	6,534,758	2628	40	371	6	108,214	574	530	208	192
Arauca	265,166	1890	713	200	75	6805	320	4703	149	2190
Atlántico	2,489,425	6866	276	408	16	43,185	2153	4986	277	641
Bogotá, D.C.	7,979,839	0	0	0	0	121,756	464	381	149	122
Bolívar	2,121,938	2010	95	266	13	41,814	213	509	49	117
Boyacá	1,278,156	414	32	120	9	21,055	51	242	25	119
Caldas	989,928	351	35	99	10	15,614	75	480	34	218
Caquetá	483,848	1171	242	257	53	11,179	378	3381	200	1789
Casanare	362,730	3994	1101	315	87	7345	498	6780	182	2478
Cauca	1,391,737	367	26	59	4	27,922	103	369	33	118
Cesar	1,041,190	1678	161	283	27	22,356	545	2438	219	980
Chocó	505,052	66	13	5	1	13,207	11	83	3	23
Córdoba	1,736,115	3393	195	263	15	37,200	1034	2780	194	522
Cundinamarca	2,721,398	5351	197	331	12	49,334	310	628	135	274
Guainía	42,127	15	36	2	5	1066	1	94	1	94
Guajira	985,392	725	74	97	10	26,748	208	778	64	239
Guaviare	112,629	217	193	18	16	2970	14	471	5	168
Huila	1,168,863	7080	606	951	81	23,224	1428	6149	687	2958
Magdalena	1,272,549	3279	258	292	23	27,768	866	3119	184	663
Meta	979,735	4376	447	636	65	19,117	1083	5665	551	2882
Nariño	1,765,893	103	6	24	1	33,528	16	48	5	15
Norte de Santander	1,367,676	10,566	773	1595	117	25,705	2928	11,391	1068	4155
Putumayo	349,612	550	157	118	34	8158	112	1373	52	637
Quindío	568,516	413	73	29	5	9283	81	873	17	183
Risaralda	957,245	1334	139	147	15	15,231	196	1287	85	558
San Andrés	77,101	1151	1493	65	84	1299	34	2617	2	154
Santander	2,071,011	10,439	504	547	26	33,006	1428	4326	435	1318
Sucre	859,955	1661	193	120	14	17,232	451	2617	69	400
Tolima	1,412,205	7280	516	884	63	25,513	992	3888	437	1713
Valle del Cauca	4,660,896	27,861	598	1114	24	73,760	3307	4483	828	1123
Vaupés	44,081	18	41	0	0	1249	4	320	1	80
Vichada	73,715	79	107	5	7	2093	13	621	1	48
Total	48,747,574	108,087	222	9802	20	876,093	19,963	2279	6365	727

^a^ *LC* Laboratory-confirmed

### Geographic distribution of ZVD cases

**Fig. 2:**
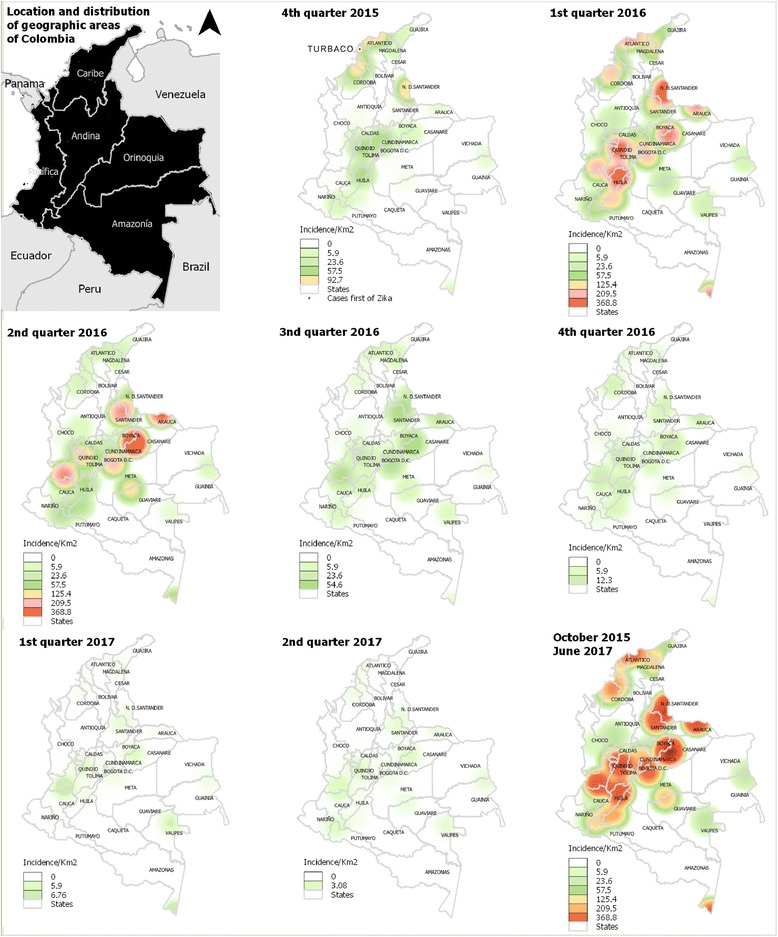
Geographically distribution of Zika cases in Colombia (October 10th, 2015-June, 24th 2017) using Kernel density estimator maps

The Kernel density map (Fig. 2) shows the areas with high concentration of cases during each quarter of each year and throughout the study period (October 2015–June 2017). In 2015, cases were concentrated in two geographic regions of the country; in the Caribbean region, mainly in states of San Andrés and Providencia Islands (not included in the map), Magdalena, Sucre, Atlantico, Bolívar (the state where the first Zika cases appeared in October 2015) and Córdoba. Another affected area was the Andean region, mainly in the state of Norte of Santander, where the intensity was higher, compared to Cundinamarca, Huila and Tolima.

In 2016, all geographic regions of the country showed at least one state with high concentration of cases, the Andean region and Orinoquia were the most affected during the four quarters (Fig. 2). In the first quarter of 2016, the cases were concentrated in four states in the Caribbean region (cluster in San Andres and Providencia, Atlantico, Magdalena and Córdoba), four in the Andean region (cluster in Norte de Santander, Huila and Tolima, Santander), 3 in the Orinoquia region (cluster in Casanare and Arauca, Meta), 2 in the Amazonia region (Cluster in Amazonas and Caqueta) and 1 from the Pacifica region (Valle del Cauca) (Fig. 2). In the second quarter of 2016, the number of clusters is decreased in comparison with the previous quarter, so clusters were located in Casanare, Arauca, Valle del Cauca and Santander. In the same quarter the number of ZVD cases also decreased in several areas, however, in Meta, Guaviare, Vichada, Quindio and Cauca an increased was seen. Between the third quarter of 2016 and the second one of 2017, the intensity of cases decreased in the whole country.

In general, throughout the study period (October 2015–June 2017), the states San Andrés and Providencia, Casanare, Norte de Santander, Arauca, Huila, Valle del Cauca, Tolima, Santander, Amazonas, Meta, Atlantico, Magdalena, Caquetá, Cundinamarca, Córdoba, Sucre, Guaviare, Cesar and Putumayo were most affected by ZVD in Colombia (Fig. 2).

### Functional relationship between ZVD indicator and GBS cases

**Fig. 3:**
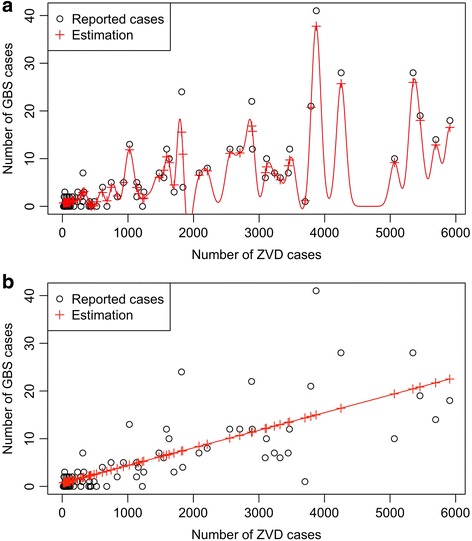
**a** Prediction of the number of Guillain-Barré Syndrome (GBS) cases given the number of Zika virus disease cases (ZVD) using kernel ridge regression and Gaussian Kernel, Colombia, October 10th, 2015-June, 24th 2017. **b** Linear regression for predicting the number of GBS cases given the number of ZVD cases, Colombia, October 10th, 2015-June, 24th 2017

Figure 1 suggests that there is a relationship between GBS and ZVD cases in the country due to GBS cases started happening more frequently when ZVD cases increased, however, Fig. 3a and b shows that there is no proportional relationship between the number of GBS and ZVD cases, e.g., in the third week of 2016 were reported 3878 and 41 of ZVD and GBS cases, respectively, and in week #7, the number of reported GBS cases drop down to 14 cases, although the number of ZVD cases increased to 5695. Instances, like this one, evidence that the increasing of ZVD cases does not imply more GBS cases. Other times the more ZVD cases take place, the more GBS cases happen as well, e.g., in the second week of 2016 2888 and 22 of ZVD and GBS cases respectively were reported, and both increased in the third week until 3878 and 41 of ZVD and GBS cases, respectively.

From the geometrical point of view, a straight line does not fit the data points (Fig. 3a and b). Figure 3a shows the estimation of the number of Guillain-Barre cases given the number of ZVD cases. This prediction has been estimated through the kernel ridge regression and the Gaussian Kernel. Its accuracy, measured in terms of Root Mean Square Error (RMSE) and the coefficient of determination are 1.77 and 80.7%, respectively. In Fig. 3a, both the red line and crosses correspond with the estimation (or prediction) while the circles correspond with the actual cases. The red crosses (and the line) are very near to the circles, even some crosses overlap circles. This means the prediction has a low error, i.e., the prediction model is very accurate.

On the other hand, Fig. 3b. shows the linear-regression-based estimation of the number of GBS cases given the number of ZVD cases. The linear regression accuracy in terms of RMSE and the coefficient of determination are 4.87 and 58.53%, respectively. The straight line and crosses shown in Fig. 3b are farer from the circles than in Fig. 3a. This means the linear model has underfitting (is less accurate).

During the cross validation, the RMSE achieved for the second order polynomial kernel, the linear kernel, the sigmoid kernel, and the Gaussian kernel are 9.15, 9.2, 10.7, and 7.2 respectively. Finally, the best setting for the Gaussian kernel is where the regularization parameter and radius are equal to 5 × 10–3 and 10–4, respectively, where its coefficient of determination and RMSE over the whole dataset are 0.95 and 0.96, respectively.

## Discussion

This study provides the first general analysis of ZVD in Colombia in the period from October 2015 to June 2017. The paper shows that Zika virus disease has had a huge impact on public health for the numbers of people affected and the presentation of Guillain-Barre syndrome and microcephaly cases associated to Zika virus disease. This study updates the epidemiological analysis of the Zika virus disease situation, it describes the geographical distribution of Zika virus disease and shows the numerical relationship between Zika virus disease with microcephaly cases and Guillain-Barre syndrome.

According to the retrieved data Zika’s attack rate among the total population of the country was 222 ZVD cases per 100,000 populations. This attack rate is similar to that observed in other Latin American countries such as Brazil, Venezuela, Costa Rica and Salvador [20]. Among the pregnant population, higher attack rates compared with total population were observed, but this situation has already been described previously in Colombia [21] and may be associated with an enhanced surveillance in this population sub-group due to congenital malformations that produces the diseases, such as microcephaly and other congenital malformations of the central nervous system.

Similar to other countries (e.g., Brazil) [22, 23], the most affected population in Colombia were women (AR, 286 per 100,000 population) and people between 20 and 39 years old (AR, 305 to 375 per 100,000 population). Pregnant women (18.5%), and people older than 65 years (3.18%) are considered as groups at risk by INS [14], because their post-infection condition might be more complicated than other groups. On the other hand, the rest of the affected population includes the economically active population, and thus it might be costly for the nation [24]. We recommend to carry out another study including the disability adjusted life years (DALYs) needs to be calculated in the near future.

The increasing number of both GBS and microcephaly cases due to ZKD has been described [7, 8]. By 24th June 2017, 0.22% (AR, 222 ZVD cases per 100,000 population) of Colombian population has been affected by ZVD [16], including almost 19,963 cases of infected pregnant women. This is an alarming situation because congenital defects increase during ZVD transmission, as has been observed in Colombia, Brazil and other regions of America [21]. The number of cases confirmed in laboratories is lower (9.10%) than reported in other countries [3]. Most cases confirmed in laboratories correspond to those notified in pregnant women (64.94%). During pregnancy the infection with ZIKV is associated to microcephaly and other abnormalities of central nervous system [25].

In March 2016, five months after the Zika virus outbreak, microcephaly cases were 1156 increased, which means that cases of microcephaly may correspond to congenital 1157 infections of women infected by ZIKV. Currently (June 2017, epiweek 25) Colombia has 710 ZVD cases associated with microcephaly; 174 were confirmed by laboratory. The increase of microcephaly cases during ZIKV transmission represents new information for clinicians because this active surveillance just started to be carried out after the first confirmed cases in Brazil. Prior to 2016, In Colombia, surveillance of microcephaly cases and other abnormalities of central nervous system, included evaluation for pathogens of the STORCH complex (syphilis, toxoplasmosis, other agents, rubella, cytomegalovirus, and herpes) [26]. ZIKV antibody testing was not done in Colombia due to high cross-reactivity with other endemic flavivirus [26].

The cases of ZVD in Colombia are decreasing, nevertheless, it is possible that new cases of microcephaly associated to ZVD will be present in the next few months when currently infected pregnant women deliver their new babies. Currently, CDC and INS are collaborating to understand long-term effects of Zika virus infection during pregnancy in Colombia, this surveillance will track for 5 years 5.000 pregnant women, as well as their spouses and children [27]. On the other hand, Zika virus also affects the hearing, visual and cognitive capacities of new-borns [28] and in Colombia this level of impact remains unknown.

Currently with the emergence of Chikungunya, Dengue and Zika a new challenge of differential diagnosis must be faced in the country, however, this emergence will present a high cost for the public health system of Colombia [1]. Geographically, the most affected states correspond to the Caribbean, Andean and Orinoquía regions of Colombia.

The Zika virus seems to have spread from the Caribbean area towards the Andean region in Colombia. It is interesting to note that there are hotspots in Norte de Santander, reporting area that are close to the Venezuelan border, where commercial activity and border crossing is intense. We believe that the high impact of ZIKV in Colombia was influenced by people moving across borders, because it is possible that people moving throughout the borders increased the mobility of the virus. We should remember that Colombia after Honduras y Belize is the country with the third highest density of ZIKV-infected people [3]. With the exception of areas of the country at 2000 m above sea level (i.e. Bogota, DC), all the 32 Colombian states were affected, the spread of the virus affected small, medium and large provinces; some cases were located at or more than 2000 m above sea level, despite the absence of known vectors for ZIKV at high elevation, these high altitude infections may be explained by travel to endemic areas or sexual transmission.

Several studies report that Guillain-Barre is associated with several etiologies, including ZIKV [1]. In this study, we have adopted kernel ridge regression to predict the number of Guillain-Barre cases given the number of ZVD cases. This model is appropriate where functional dependence is not linear (i.e., it is not proportional), in fact, Fig. 3a and b, shows there is no proportional relationship between the number of GBS and ZVD cases. Instances, like this one, evidence that the increasing of ZVD cases does not imply more GBS cases. It is important to emphasize that the linear kernel correspond to classical linear regression model, and there are two kernel which correspond with the more accurate models than the linear one. These outcomes are coherent with the distribution of the data points which cannot be aligned in a straight line in Fig. 3, which also means that there is no proportional relationship between ZVD and GBS cases, as it was above mentioned. As a consequence, the Gaussian kernel is the best choice for the prediction because with this kernel, KRR reported the smallest RMSE during cross validation, i.e., this is the most accurate kernel for this domain. We have performed 5-fold cross validation for tuning parameters (i.e. the radius and regularization parameters for the Gaussian kernel) and choosing the most appropriated kernel between sigmoid, polynomial, and Gaussian kernel. The cross validation results reveal that the regression model with the latter kernel outperforms the others in terms of accuracy.

The above mentioned regression model accurately predicted the number of Guillain-Barre cases. Nevertheless, there is an over fitting risk due to small sample size compared with those available in other domains. However, we lack another independent dataset to carry out the test, so the best setting achieved through cross validation was tested over the only dataset available to us. So in this study we provide evidence which might confirm the link between both diseases, notwithstanding that there are several reports where GBS has multiple associated etiologies.

As this incidence of ZVD decreases, GBS cases are expected to decrease as well. However, because of multiple etiologies, we expect GBS to persist even when ZVD disappears. In fact, we showed that the functional relationship between both diseases is not proportional, so it is not accurate to state the more Zika cases appear, the more Guillain Barre cases shall take place. Instead, as it is shown in Fig. 3a and b, the more Zika cases happen, the Guillain Barre cases will either increase or decrease, otherwise the linear kernel (i.e., the classical linear regression model) would have outperformed other kernels. Nonetheless, the prediction model adopted in this work is able to capture the regular patterns accurately, in spite of there are other known and latent (hidden) factors which explain the relationship between both illnesses. The drawback of KRR is that this model does not allows us interpret the prediction, however, it is the most accurate in this context.

## Conclusions

This work contributes with an update on epidemiological analysis of the situation of ZVD in Colombia. The study describes the geographical distribution of ZIKV infection in the country and shows the association with microcephaly cases and GBS. A kernel ridge regression may be important to predict the number of Guillain-Barre cases given the number of ZVD cases. This study provides evidence that confirms the relationship between both diseases. Zika virus may have some influence of the new presentation of GBS in Colombia. There is a public health concern about the future of microcephaly cases in Colombia. Data essential in ZIKA control such as disaggregated data by age-group and by altitude are currently missing.
